# The Role of Dairy Products in Healthy Weight and Body Composition in Children and Adolescents

**DOI:** 10.2174/157340111794941111

**Published:** 2011-02

**Authors:** Lisa A Spence, Christopher J Cifelli, Gregory D Miller

**Affiliations:** 1 American Dietetic Association, Chicago, Illinois, USA; 2 National Dairy Council, Rosemont, Illinois, USA; 3 Dairy Research Institute, Rosemont, IL, USA

**Keywords:** Dairy, calcium, body weight, body composition, body fat.

## Abstract

Overweight and obesity are major public health concerns with approximately 32% and 17% of U.S. children aged 2 – 19 being classified as overweight or obese, respectively. While the cause of overweight and obesity is multi-factorial, changes in eating habits and physical activity patterns have been proposed as contributing factors to the obesity epidemic. For example, the displacement of nutrient rich foods and beverages with non-nutrient dense items may be influencing childhood obesity. Many children do not consume the recommended servings of the Food Groups to Encourage, i.e. low-fat and fat-free dairy foods, fruits, vegetables, and whole grains identified by the 2005 Dietary Guidelines for Americans which results in low intakes of calcium, potassium, fiber, magnesium, and vitamin E. While attention has focused primarily on reducing energy intake and/or increasing energy expenditure for weight maintenance, a promising beneficial role for dairy products in weight management has emerged. Most research has focused on adults, but there is evidence in children and adolescents indicating either a beneficial or neutral effect of dairy food consumption on body weight or body composition. The current review provides and assessment of the scientific evidence on the effects of dairy food consumption on body weight and body composition in children and adolescents.

## I INTRODUCTION 

Overweight and obesity are global public health concerns [1, 2]. Data from National Health and Nutrition Examination Surveys (NHANES) 2007-2008 indicates that 31.7% of US children aged 2 – 19 years are overweight, with approximately 17% classified as obese [3]. Similarly, the Early Childhood Longitudinal Study shows obesity prevalence at 18.4% among 4-year-old US children with the highest rates in American Indian/Native Alaskan, Hispanic, and non-Hispanic black children [4]. If the current trends continue, the prevalence of obesity in children and adolescents would almost double by 2030 [5].

Obesity in children and adolescents is of great concern because excess body fat increases the risk of premature death, coronary heart disease, type 2 diabetes, hypertension, stroke, some types of cancer and other debilitating conditions [6]. Data from the NHANES III demonstrates that 4.2% normal weight adolescents and 28.7% overweight adolescents have metabolic syndrome suggesting that more than 900,000 adolescents nationwide have metabolic syndrome [7]. A more recent report indicates metabolic syndrome prevalence among overweight children aged 8-11 years at 6.5% to 9.5% and among overweight adolescents aged 12-14 years at 27% to 44% [8]. Furthermore, maintaining a healthy body weight throughout childhood may help reduce the risk of becoming an overweight or obese adult [9-12]. For example, Whitaker and colleagues estimated that nearly 80% of children who were overweight at age 10-15 years were obese at age 25 years [11]. Similarly, Freedman and colleagues showed that 25% of obese adults were overweight as children [12]. Therefore, identifying dietary patterns and/or components of the diet that help children and adolescents maintain a healthy body weight is of utmost importance. 

Because the cause of overweight and obesity is multi-factorial, successful prevention or treatment depends on multiple integrated actions. For overweight children and adolescents, the goal is to reduce the rate of weight gain while achieving normal growth and development [9]. Although attention has focused primarily on reducing energy intake and/or increasing energy expenditure, a promising beneficial role for dairy products in weight management has emerged. In recent years, a body of scientific evidence of human clinical, observational and animal model studies has evolved that supports a relationship between the consumption of dairy foods and weight management in adults [13-15]. While there is less research in children and adolescents investigating the relationship between dairy and weight/body composition, in general, studies indicate either a beneficial or neutral effect of dairy foods or calcium consumption on body weight or body composition. The current review provides an assessment of the scientific evidence on the relationship between dairy food consumption and body weight and body composition in children and adolescents. First to be discussed are cross sectional analyses, followed by prospective observational studies, and finally randomized clinical trials. 

## II OBSERVATIONAL STUDIES 

### 1 Cross Sectional Analyses

Analyses of cohorts of children from various countries and of different racial and ethnic groups in the U.S. have demonstrated an inverse relationship between milk or dairy intake and indices of body weight and/or body fat. Table **1** presents the cross-sectional studies that have examined the association between milk and/or dairy intake and BMI, body weight and/or body fat in children and adolescents [16-38]. A cross-sectional study in 884 children from Southern Italy utilized data from a lifestyle and diet survey to evaluate the association between body fat and body weight and frequency of milk consumption [22]. After controlling for confounders, linear regression analysis of the frequency of consumption of milk in relation to age- and sex-specific BMI z-scores showed an significant inverse association. Milk consumption remained significantly and inversely associated with BMI z-score in the whole-milk consumers when controlling for age and the frequency of consumption of various foods; however, this association was no longer significant when children consuming skimmed milk were included in the analysis. The authors note that this is the first report showing a significant inverse association between frequency of milk consumption and BMI in children [22]. In a cross-sectional analysis, researchers evaluated dietary calcium intakes, anthropometric measures, and bone health in 50 pre-pubertal children from New Zealand who had a history of avoiding milk consumption [18]. This cohort of 50 children was compared to a cohort of 200 children who drank milk. The milk avoiders were shorter, had smaller skeletons, lower total-body bone mineral content, and lower z-scores for areal bone mineral density than did the control children of same age and sex from the same community. Furthermore, the milk avoiders had higher BMIs than the milk consumers and the prevalence of obesity in the milk avoiders was 18%. These data suggest that in growing children, long-term avoidance of cow milk is associated with small stature, poor bone health, and may be related to obesity [18]. A cross-sectional analysis of baseline data from the Female Adolescent Maturation study including 323 Asian and Caucasian young adolescents aged 9 to 14 years from Hawaii demonstrated that calcium intake, age, and physical activity were significantly inverse association with iliac skinfold thickness [20]. Calcium from dairy sources had a stronger association than total calcium intake, while non-dairy calcium was not associated with weight or iliac skinfold thickness. One milk serving was associated with a decrease of 0.78 mm in iliac skinfold thickness, while soda intake had a significantly positive association with weight. Additionally, the inverse association between dairy and iliac skinfold thickness was stronger in Asian versus Caucasian adolescents. Thus, the researchers concluded that decreasing soda and increasing dairy consumption among Asians may help maintain body fat and weight during adolescence [20]. In an observational case-control study of 53 Puerto Rican children, frequency of fruit juice consumption, hours of daily TV viewing, maternal BMI, and lower dairy product intake were associated with obesity. A significant negative correlation existed between dairy product consumption and BMI with a 59% reduction in obesity risk associated with high dairy product consumption [16]. In a sample of 1701 children from 3rd to 7th grade in schools from three geographical Chilean regions, there was a significantly inverse association between obesity and dairy product consumption [21]. In a study of 365 Argentinean children with average age of 10 years, increased milk consumption was associated with lower waist circumference, systolic blood pressure, and insulin resistance [34]. 

Several studies utilizing U.S. national data sets have evaluated the association between milk or dairy intake and indices of body weight and/or body fat. LaRowe *et al.* [27] utilized a food-based approach to identify predominant beverage intake patterns among preschool children aged 2 to 5 years and school-aged children aged 6 to 11 years from NHANES 2001-02. Children from both age groups classified as high-fat milk consumers had a significantly greater micronutrient intakes as compared to all other beverage clusters. Total energy intake from beverages in children classified as high-fat milk consumers was higher as compared to all other beverage clusters. However, BMI was significantly lower in the high-fat milk cluster as compared to the water, sweetened drink, and soda clusters for children aged 6 to 11 years and a trend existed in children aged 2 to 5 years. In general, the children in the high-fat milk cluster had the lowest BMI, but among the best overall diet quality as reflected by the HEI and micronutrient intakes, which the authors concluded was support that children with better diet quality would have lower BMIs [27]. Another analysis of NHANES data demonstrated that children with higher milk intakes have better nutrient intakes and there is no negative impact on BMI [31]. In this analysis, intakes of vitamin A, calcium, phosphorus, magnesium, potassium, and saturated fat were higher in milk drinkers (including flavored and plain milk), than milk nondrinkers. Additionally, the BMI measures of milk drinkers were comparable to or lower than measures of nondrinkers [31]. Moore and colleagues explored the association between dairy consumption and body fat among more than 10,000 U.S. children and adolescents participating in two of the NHANES from 1988-1994 and 1999-2002 [30]. Results showed that, in both survey periods, a low dairy intake (< 1serving/day for girls and < 2 servings/day for boys) among 12-16 year olds was associated with a higher BMI and greater body fat. Additionally, similar results were seen when examining total calcium intake in relation to body fat among the same age range. However, among younger children, aged 5 to 11 years, there was no consistent association between dairy or calcium intake and body fat levels [30]. In the Growing Up Today Study, overweight participants consumed fewer dairy products than non-overweight youths. Furthermore, the reported consumption of dairy servings was less in overweight participants than mean servings for the entire cohort [17]. Recently, Bradlee and colleagues analyzed data from NHANES III to assess the association between food group intake and central body fat in children and adolescents [38]. While there was no association between dairy intake and central obesity in children, mean dairy intake was inversely associated with central obesity in adolescent boys and girls [38]. 

While the above mentioned studies have reported inverse relationships between milk and/or dairy intake and indices of body weight and/or body fat, there are studies in which no association has been found. In a descriptive and multivariate regression  analysis  of children and adolescents aged 6 to 19 years participating in the U.S. Department of Agriculture’s Continuing Survey of Food Intakes, 1994-96, 1998, BMI was not associated with consumption of milk, regular soft drinks, regular or diet fruit drinks, or non-citrus juices. For girls, BMI was negatively correlated with milk consumption and positively correlated with diet carbonated beverages, but these relationships were weak [19]. Fiorito *et al*. [25] assessed the relationship among girls’ weight status, dairy intake, and total energy intake. Using 24-hr recalls and energy calculations, 172 girls (11.3 ± 0.3 years old) were classified as under-reporters, plausible reporters, or over-reporters. When all subjects were analyzed, consumption of the recommended amount of dairy (3 servings/d) was significantly associated with lower weight status and percent body fat. However, when under-reporters, plausible reporters, or over-reporters were analyzed separately, there was no association between dairy intake and body weight or percent body fat [25]. In another study, there was no association between the amounts of total beverage, milk, 100% fruit juice, fruit drink or soda consumed and weight status. Furthermore, there was no significant association between the types of milk (based on percent fat) consumed and weight status [26]. Keller *et al*. examined whether increased sugar-sweetened beverage intake was associated with decreased milk/calcium intake and body weight from previously collected, laboratory-based meal data from twins [33]. The results showed that the intake of sugar-sweetened beverages (e.g. cola, juice and juice drinks) were negatively associated with the intake of milk, calcium and vitamin D; however, neither calcium nor milk intake was associated with BMI or waist circumference [33]. More recently, Wiley analyzed NHANES data from 1999 – 2004 to examine the relationship between milk and dairy product consumption and BMI among children 2 – 4 and 5 – 10 yr of age [35]. Young children in the highest quartile of milk intake had higher BMIs than all lower quartiles. Among children 5 – 10 year of age, dairy intake was not related to BMI; however, those in the highest quartile of milk intake had higher BMI than those in the second quartile. The author concludes that milk consumption is positively associated with BMI, especially in children 2 – 4 yr of age [35].

In addition to the studies that have examined dairy and/or milk consumption, some studies have assessed relationship between calcium intake and adiposity. For instance, a cross-sectional analysis of 96 male and female post-pubertal adolescents compared the calcium intake of normal weight and obese adolescents [28]. The mean calcium intake was lower in the obese subjects as compared to the normal weight subjects (585.2 ± 249.9 vs. 692.1 ± 199.5 mg/day, respectively) and calcium intake was inversely related to trunk adiposity, circulating insulin levels, and insulin resistance. When calcium intake was analyzed via quartile analysis, girls in the highest quartile displayed the greatest decrease in adiposity and insulin resistance. The authors conclude that calcium intake is negatively associated with body fat and insulin levels, especially in obese girls [28]. In addition, Palacios* et al.* [28] examined the relationship between calcium-rich food consumption and body weight/BMI in adolescents aged 13 to 18 years and reported that calcium intake was negatively associated with BMI in boys aged 13 – 15 years. However, this correlation was not found among older boys or in girls of any age [28]. Recently, Tylvasky and colleagues examined the association between calcium intake and fat mass, lean body mass, bone mineral content and body mass index (BMI) in African-American children and adolescents [37]. Analysis of the food records indicated that less than half the participants met the Dietary Reference Intake for calcium, magnesium, phosphorus, potassium, iron, zinc, folate, riboflavin, vitamin B_12_, vitamin C, vitamin D, vitamin A or vitamin K. Participants in the lowest calcium intake group had the lowest energy-adjusted intakes of fiber, riboflavin, folate, vitamin A, vitamin D, magnesium, phosphorus, potassium, iron and zinc. After adjusting for confounding variables, there was no association between calcium intake and fat mass, lean body mass, bone mineral content or BMI in the whole sample. However, gender-specific analyses showed that those females in the lowest calcium-intake group had a higher fat mass, a higher percent fat mass, and a lower lean body mass than those in the middle and highest calcium-intake groups. The results of this study showed that African American girls with the lowest intakes of calcium had increased body fat and lower vitamin and mineral intakes as compared to those girls with the highest intakes of calcium [37]. 

### 2 Prospective Studies

Table **2** presents the prospective studies that have investigated the relationship between dairy intake and BMI, body weight, and body fat [39-51]. Skinner and Carruth published two reports evaluating the effects of preschool food consumption on body composition, specifically dietary calcium’s relationship to body fat, in a cohort of 50 children who were followed from two months to 8 years of age [39,40]. In regression analyses, longitudinal intakes of calcium, dairy foods, and monounsaturated fat were significantly negatively related to body fat at 70 months. In a regression analysis of dietary calcium and polyunsaturated fat intake and percent body fat at 8 years of age, dietary intakes of these nutrients were negatively related to percent body fat. Also, calcium intake was positively related to dietary variety, while negatively related to carbonated beverages and other sweetened beverages. The authors recommend that children should be strongly encouraged to regularly include calcium-rich foods and beverages in their diets, specifically skim, 1% or 2% fat milk and other low-fat dairy products [39,40].

Rockell *et al*. [44], in a follow up to the earlier report by Black et al. [18], demonstrated that milk avoiders had lower calcium intakes and were shorter in stature, with elevated BMI, poor skeletons and lower z-scores for both areal bone mineral density and volumetric bone mineral density, compared with a reference population of milk drinkers. Using data from the Framingham Children’s Study, Moore *et al*. [46] estimated the effect of dairy intake in early childhood on the acquisition of body fat throughout childhood. The results showed that the consumption of <1.25 servings of dairy/day in girls and <1.70 servings of dairy/day in boys during early childhood was associated with increased risk for gaining excessive amounts of body fat by early adolescence. In addition, girls that consumed calcium levels that were in the lowest tertile gained an extra 25 mm of subcutaneous fat by early adolescence. Interestingly, the observed protective effect of dairy was not explained by calcium and magnesium levels. The results of this study support the hypothesis that low-intakes of dairy products in early childhood may promote the acquisition of body fat over time [46]. 

While the above prospective studies have indicated inverse associations between dairy and/or calcium intake and body weight and/or body fat, there are other prospective studies in which no associations have been noted. Analysis of data from the Growth and Development Study showed that there was no relationship between BMI z-score or percent body fat and dairy or calcium consumption over time [41]. The authors conclude that the results refute the idea that dairy foods should be avoided during adolescence to avoid excess body weight and/or body fat accumulation [41]. To examine the effects of calcium and dairy intake on body weight, Berkey *et al*. [45] analyzed food frequency questionnaires from adolescents that participated in the longitudinal Growing Up Today Study. The results showed that baseline calcium intake was 1291 mg/day and 1145 mg/day for boys and girls, respectively, and that milk intake declined with age in both groups. In addition, the results indicated that adolescents that drank ≥ 3 servings of milk/day had significantly larger BMI gains from year-to-year than those who drank between 1 – 2 servings of milk/day. However, after adjusting for energy intake, the association between milk consumption and BMI was not statistically significant. Finally, dairy fat was not a strong predictor of weight gain in adolescent boys or girls as compared to other types of fat [45]. 

Barr [48] conducted a post-hoc analysis to determine whether habitual calcium intake was an independent predictor of percent body fat. The analysis was performed on data obtained from 45 peripubertal girls who took part in a two-year prospective study that examined the effects of calcium on bone mineral density. Girls below the median intake for calcium from all foods (i.e. below 773 mg/d) had higher baseline body weight, total fat mass, percent body fat, and percent trunk fat as compared to girls above the median intake. However, when the Eating Attitudes Test was included in the analysis as a covariant there were no apparent statistical differences at baseline. Moreover, calcium intake was not associated with changes in body weight or composition over the 2 years. The author concluded that the results suggest that the inverse association between calcium and/or dairy intake and body weight changes may result from unmeasured variables and reverse causation (i.e., heavy girls are more likely to exclude dairy from their diets) [48]. In a cohort of 192 non-Hispanic white girls, calcium intake was evaluated from ages 5 to 9 years as a function of mother-daughter beverage choices and as a predictor of bone mineral status [43]. Girls who met calcium recommendations had significantly greater BMD than those who did not meet recommendations. Moreover, girls who met calcium recommendations were not heavier but had higher energy intakes than did the girls who consumed less than the recommended amounts [43].

Fiorito *et al*. assessed whether beverage intake at age 5 years predicted energy intake, adiposity and weight status across childhood and adolescence [50]. At age 5 years, girls were categorized as consuming <1, ≥1 and <2, or ≥2 servings of sweetened beverages per day. The authors reported that sweetened beverage intake at age 5 years, but not milk or fruit juice intake, was positively associated with adiposity from age 5 – 15 years [50]. Recently, Huh and colleagues examined the relationship between the quantity and type of milk consumed at age 2 years with adiposity at age 3 years in preschool-aged children [51]. After controlling for confounders, higher intakes of whole milk at age 2, but not reduced fat or 1%/nonfat milk, was negatively associated with BMI at age 3. However, when the analysis was restricted to children with a normal BMI at age 2 yr, the association was attenuated. Intake of milk at age 2 yr, whether full- or reduced-fat, was not associated with the risk of incident overweight at age 3 yr. Further, neither total milk nor total dairy intake at age 2 yr was associated with BMI or incident overweight at age 3 yr. The results of this study suggest that neither the type nor the amount of dairy consumed adversely affects body weight in preschool-aged children [51].

In summary, while most of the data is generated from cross-sectional studies, the results from nearly all of the observational studies demonstrate either a beneficial or neutral relationship between the consumption of dairy and/or calcium and body weight and body composition in children and adolescents. 

## III RANDOMIZED CLINICAL TRIALS

To date, few clinical trials have been conducted to specifically investigate the effect of dairy consumption on body weight and body composition changes in children and adolescents. Most randomized clinical trials were originally designed to assess the effect of calcium supplements or a form of calcium derived from milk on bone health. A recent systematic review and meta-analysis of 17 placebo-controlled randomized clinical trials determined whether calcium supplementation in healthy children affects body weight or body composition [52]. The results of the meta-analyses showed that there were no statistically significant effects of calcium supplementation on weight, body fat, or lean body mass in healthy children [52]. 

Table **3** presents the clinical trials that introduced milk or dairy into the diets of children and/or adolescents to examine effects on body composition and weight changes [53-63]. In a randomized clinical trial of 80 healthy Caucasian girls, subjects were randomized into milk or control groups stratified by pubertal stage to investigate the effect of milk on total body mineral acquisition, height, weight, and lean body mass [54]. The milk group consumed one pint (598 mL) of whole or reduced fat milk each morning for 18 months while the control group followed their habitual diet which included 150 mL of milk per day. The milk-supplemented group experienced a calcium intake increase from 739 mg/day to 1125 mg/day and significant increases in dietary protein, phosphorus, magnesium and zinc over the 18 month study and a trend towards increased energy intake. Bone mineral content and bone mineral density significantly increased more over 18 months for the milk group as compared to the control group. There were no significant differences in changes in height, weight, lean body mass and fat body mass in either group. However, a trend in the milk group was demonstrated as a greater gain in lean body mass with concomitant reduction in percent body fat and an overall gain in weight [54].

Chan *et al.* [53] investigated the effect of calcium supplementation with dairy products on the bone and body composition of pubertal girls in a randomized control study. Forty-eight Caucasian girls were randomized to a diet supplemented with dairy products including 2% milk, fat-free milk, 2% chocolate milk, American cheese, and regular fruit yogurt with total calcium at 1200 mg calcium daily or to the control diet which was the subjects’ usual diet. While the dairy group had higher intakes of calcium, phosphate, vitamin D, and protein than control subjects, the fat, saturated fat, and energy intake were not different between the two groups. The dairy group had significantly greater increases over 12 months in bone mineral density at the lumbar spine and total body bone mineral than control subjects. There were no differences between the groups in lean body mass or percent body fat at baseline or 12 months. Chan and colleagues concluded that young girls whose dietary calcium intake was provided primarily by dairy products had an increased rate of bone mineralization and that the increased intake of dairy foods was not associated with excessive weight gain or increased body fat [53].

Lappe and colleagues analyzed data from a project that investigated the effects of a calcium-rich diet on bone health in 9 year old girls to evaluate the influence of the diet on weight gain during 2 years of the study [57]. Participants were randomly assigned to a calcium-rich diet supplying at least 1,500 mg of calcium per day (primarily from dairy foods) or their usual diet. Although girls in the treatment group consumed approximately 150 more calories per day, they did not have greater increases in body weight, BMI, or fat or lean mass compared to the control group. Girls on calcium-rich diet also significantly increased their intake of essential nutrients, including calcium, protein, vitamins A and D, phosphorus and magnesium. The researchers concluded that calcium-rich diets do not cause excessive weight gain in pubertal girls while contributing positively to overall nutrition [57].

In another randomized clinical trial, 28 boys were randomly assigned to consume, in addition to their habitual diet, 3 servings/day of 1% fluid milk or juice not fortified with calcium while engaged in a 12-week resistance-training program [56]. The milk group had significantly higher intakes of protein, fat, vitamins A and D, riboflavin, calcium, phosphorus, and magnesium, and lower intakes of carbohydrate and vitamin C. While all subjects experienced significant changes in height, sum of seven skinfolds, body mass, lean body mass, fat mass, whole body bone mineral content, and maximal strength in the squat and bench press, there were no statistical differences between the groups. The milk group had a significantly greater increase in bone mineral density compared to the juice group. Additionally, a non-significant trend of reduced body fat was observed in the boys who consumed milk compared to those who consumed juice. The authors suggest that increasing the intake of milk in physically active adolescent boys may enhance bone health and may have the potential to influence body fat [56].

In a 3 year study (2 years of supplementation and 1 year follow-up), teenage girls were able to significantly increase their bone mineral density at the trochanter, femoral neck and lumbar spine when supplemented with dairy foods to a mean calcium intake of 1160 mg/day [55]. Bone mineral content, particularly at the trochanter and to a lesser extent at the lumbar spine, also increased. While the supplementation with dairy foods did have a beneficial effect on bone health, no adverse effect was evident on body weight, fat and lean mass or blood lipid profiles [55]. In a 16-week randomized controlled trial, 98 overweight and obese Chilean children who habitually consumed sugar-sweetened beverages were randomly assigned to a control group who continued regular beverage consumption versus an intervention group who received three servings of flavored milk per day [60]. The intervention (milk) group significantly increased their consumption of milk while significantly decreasing their consumption of sugar-sweetened beverages. The control group’s milk consumption did not change but consumption of sugar-sweetened beverages significantly increased. There were no differences between the groups in percent body fat; however, a significant increase in lean body mass occurred in the milk group [60]. 

In another 16-week randomized clinical trial, 45 overweight children were randomly assigned to milk supplementation at four servings per day versus three servings of sugar sweetened beverage per day within a healthy diet plan to assess effects on body weight, body composition, and metabolic variables [62]. The children receiving 3 servings of sugar sweetened beverages per day also received 4 servings skim milk per week and 5 servings of 1% chocolate milk per week; thus, an average of 1.3 servings of milk per day. All children were instructed to follow the Stoplight meal plan but were not given any specific energy restriction guidelines. There were no differences in body weight, body composition, or metabolic variables between the groups. However, in the high milk consumption group, children experienced a decrease in insulin response after an oral glucose tolerance test indicating that milk may protect against insulin resistance [62]. Similarly, Kelishadi et al. conducted a randomized controlled trial to determine the long-term effects of a dairy-rich diet on abdominal obesity and components of the metabolic syndrome in obese prepubescent children [63]. In addition to attending 6 consecutive monthly family-centered education sessions about a healthy lifestyle, 120 obese children were randomly assigned to a control diet (i.e. no further recommendations), an isocaloric dairy-rich diet (>800 mg calcium/d) or a calorie-restricted diet (-500 kcal/d) for 3 year. In all groups, BMI-standard deviation scores and waist circumference significantly decreased after the 6-month trial. While there was a sustained rise during the follow-up period until the end of the study, the rise in BMI and waist circumference was significantly lower in the dairy group as compared to the other groups. In all groups, serum triglycerides, insulin levels, serum HDL and insulin resistance improved after the 6-month intervention. In the dairy group, the serum triglycerides, insulin levels and insulin resistance remained significantly better than baseline values until the 12 month follow-up. The researchers conclude that in addition to lifestyle changes, an isocaloric diet rich in dairy products may be a well-accepted regimen and can be a safe and practical strategy for weight control in young, overweight children [63].

In contrast to the above findings, Du and colleagues conducted a 2-year intervention trial in 757 Chinese girls from 9 primary schools to determine the effects of milk supplementation on bone mineralization and body composition [58]. The girls in each school were randomized into three groups: a milk supplemented group (330 mL of calcium-fortified milk), a milk + vitamin D group (330 mL of calcium-fortified milk + cholecalciferol), or a control group. The supplemented milk, when averaged over the 2 year, provided an additional 245 mg of calcium/d on top of the background calcium intake which average between 418 – 455 mg/d. Over the 2-year period, milk consumption significantly increased height, sitting height, total body bone mineral content, bone mineral density and body weight (as expressed by mean percentage changes from baseline). In addition, the subjects receiving additional vitamin D had significantly greater increases in the change in total body bone mineral content and bone mineral density [58]. 

Currently, there is little direct evidence that dairy consumption adversely affects body weight in children and adolescents. Overall, the majority of the research indicates that the effect of milk intake on body weight and body composition in children and adolescents is neutral. However, additional clinical research is needed to better understand this relationship in children and adolescents. 

## IV SUMMARY

Physiological, behavioral and environmental contributions all influence the development of obesity. From a nutritional standpoint, obesity develops because of long-term energy imbalance. In children and adolescents, some researchers have suggested that the consumption of larger portion sizes and away-from-home eating foods are major contributors to the obesity epidemic [64]. Additionally, it has been suggested that the displacement of nutrient-rich foods and beverages with nutrient-poor, energy-dense items may be adversely affecting childhood obesity [66-68]. For example, a longitudinal trial linked the increased consumption of some types of non-nutrient dense beverages, such as those containing only sugar, with weight gain and obesity in children [69]. This study reported that the odds of becoming obese increased by 1.6 for each additional serving of non-nutrient dense beverage consumed per day [69]. In contrast, milk is a good or excellent source of nine essential nutrients: calcium, potassium, phosphorus, protein, vitamins A, D, and B12, riboflavin, and niacin (niacin equivalents) to the diets of children and adolescents, which are critical for growth and development [70]. Thus, the consumption of adequate amounts of dairy foods helps children and adolescents meet their nutrient requirements and also improve overall diet quality [66,67,71]. 

Taken together, the available scientific evidence indicates that the consumption of milk and milk products do not adversely affect body weight or body composition in children and adolescents. Moreover, the majority of cross-sectional and prospective studies indicate a beneficial relationship between the consumption of milk and/or calcium and body weight and body composition in children and adolescents. Additionally, milk is the number one source of calcium, vitamin D, phosphorus, and potassium in the diets of children aged 2 to 18, which are important for good health [72]. For these reasons, the 2005 Dietary Guidelines for Americans recommends two servings of low-fat and fat-free milk or equivalent milk products (e.g., cheese, yogurt) daily for children aged 2 to 8 years and three servings for those 9 years and older [9]. Similarly, the American Academy of Pediatrics recommends that children consume three servings of dairy foods a day and that adolescents consume four servings of dairy foods a day [73]. More recently, the 2010 Dietary Guidelines Advisory Committee Report stated that the “consumption of the recommended daily amounts of low-fat or fat-free milk and milk products (2 cups for children ages 2 to 8 years, 3 cups for those 9 years and older) should be promoted” [74]. In addition, the report noted that lower intakes of milk and milk products may be associated with increased risk of cardiovascular disease, type 2 diabetes, poor bone health and related diseases [74]. Thus, while additional research is needed to better understand the relationship between dairy food consumption and weight management in children and adolescents, the current research continues to show that milk and milk products provide important nutrients to the diets of children and adolescents without adversely affecting body weight and body composition.

## Figures and Tables

**Table 1:** Cross-Sectional Studies Evaluating the Relationship Between Dairy and/or Calcium Intake and Body Weight and/or Body Composition in Children and Adolescents

Citation	Subject Characteristics	Results*

Tranasecu *et al.*, 2000	53 Puerto Rican children	↓ Dairy foods, ↑ obesity

Rockett *et al.,* 2001	Growing Up Today Study (n = 16,6882)	↓ Dairy foods, ↑ overweight

Black *et al., * 2002	50 milk avoiders and 200 control children	Milk avoiders ↑ BMI

Forshee and Storey, 2003	CSFII 1994-96, 1998(n = 3,311)	Milk intake ↔ BMI in boys
		Milk intake ↓ BMI in girls

Novotny *et al.,* 2004	Female Adolescent Maturation Study (n=323)	↑ Calcium intake, ↓ body weight and iliac skinfold thickness

Olivares *et al.,* 2004	Chilean children(n=1,701)	↑ Dairy intake, ↓ obesity (measured by BMI)

Barba *et al.,* 2005	BRAVO Project and the Ganniano Study (n = 884)	↑ Milk consumption, ↓ BMI z-scores

Dixon *et al.,* 2005	Children’s Health Project (n = 342)	↑ Calcium or dairy intake, ↓ BMI and skinfold measures in non-HC children 7-10 y/o
		↑ Calcium or dairy intake, ↔ BMI and skinfold in all HC children and non-HC children 4- y/o

Moreira *et al.,* 2005	Portuguese children(n=3,044)	↑ Calcium intake, ↓ BMI in girls
		↑ Calcium intake, ↔ BMI in boys

Fiorito *et al.,* 2006	Girls (n=172)	≥3 Serving dairy, ↓ BMI and % body fat in total sample
		≥3 Serving dairy, ↔ BMI and % body fat in plausible, under- and overreporters

O’Connor *et al.,* 2006	NHANES 1999-2002 (n=1,160)	Milk intake ↔ BMI
		Type of milk consumed ↔ BMI

LaRowe *et al.,* 2007	NHANES (n=541, 2-5 y/o; n=793 for 6-11 y/o)	High-fat milk cluster ↔ BMI in 2-5 y/o
		High-fat milk cluster ↓ BMI in 6-11 y/o

Palacios *et al.,* 2007	Adolescents (n=100)	↑ Calcium intake, ↓ BMI in boys 13-15 y/o
		↑ Calcium intake, ↔ BMI in boys 16-18 or girls

Dos Santos *et al.,* 2008	Post-pubertal adolescents(n=96)	↑ Calcium intake, ↓ body trunk fat in obese subjects
		↑ Calcium intake, ↔body trunk fat in normal weight subjects

Moore *et al., *2008	NHANES III (n=3,864, 5-11 y/o; n=2,231, 12-16 y/o)	↑ Dairy intake ↔ with indices of body fat in 5-11 y/o
	NHANES 1999-2002 (n=1,884, 5-11 y/o; 2,636 12-16 y/o)	↑ Dairy intake, ↓ indices of body fat in 12-16 y/o boys and girls

		Milk drinkers, ↔ BMI in 2-11 y/o
Murphy *et al.,* 2008	NHANES 1999-2002 (n=7,557)	Milk drinkers, ↓ BMI in 12-18 y/o boys
		Milk drinkers, ↔ BMI in 12-18 y/o girls

Goldberg* et al., *2009	Adolescents (n=107)	↑ Calcium intake, ↓ adiposity in males>
		↑ Calcium intake, ↔ adiposity in females

Keller *et al.,* 2009	Twin children (n=126)	Calcium or milk intake, ↔ BMI and waist circumference

Hirschler *et al.,* 2009	Argentinean children(n=365)	↑ Milk intake, ↓ waist circumference

		Highest quartile of milk intake, ↑ BMI than lower quartiles in 2-4
Wiley, 2010	NHANES 1999-2004 (n=1,493, 2-4 y/o; n=2,526, 5-10 y/o)	y/o
		Highest quartile of milk intake, ↑ BMI than quartile 2 in 5-10 y/o

Almon *et al., *2010	European Youth Heart Study (n=298 children; n=386 adolescents)	↑ Milk and dairy intake, ↔ body fat mass

		↑ Calcium intake, ↔ fat mass and lean body mass in all subjects
Tylvasky *et al.,* 2010	African American adolescents (n=186)	and males
		↓Calcium intake, ↑ fat mass and percent body fat in females

Bradlee *et al., *2010	NHANES III	Mean dairy intake, ↔ central obesity in 5-11 y/o
	(n=3761, 5-11 y/o; n=1803, 12-16 y/o)	Mean dairy intake, ↓ central obesity in 12-16 y/o

* For the associations listed, ↑ represents a positive association, ↓ represents a negative association and ↔ represents no association between the variables listed.

**Table 2:** Prospective Studies Evaluating the Relationship Between Dairy and/or Calcium Intake and Body Weight and/or Body Composition in Children and Adolescents

Citation	Subject Characteristics	Results*
Carruth and Skinner, 2001	Children (n=53)	↑ Calcium and dairy, ↓ body fat
Skinner *et al.,* 2003	Children (n=53)	↑ Calcium, ↓ percent body fat
Phillips *et al.,* 2003	Non-obese girls (n=196)	↑ Calcium and dairy, ↔ BMI and percent body fat
Newby *et al.,* 2004	North Dakota Special Supplemental Nutrition Program for WIC (n=1,345)	↑ Milk intake, ↔ weight change and BMI
Fisher *et al.,* 2004	Non-Hispanic white girls (n=192)	≥AI for calcium, ↔ BMI as <AI for calcium
Rockell *et al.,* 2004	Milk avoiders (n=46)	Milk avoidance, ↑ BMI
Berkey *et al.,* 2005	Growing Up Today Study (n=12,829)	↑ Calcium and milk, ↑ BMI gain (↔ when adjusted for energy)
Moore *et al.,* 2006	Framingham Children’s Study (n=92)	↓ Dairy, ↑ risk for gaining body fat
DeJongh *et al.,* 2006	Children (n=178)	↑ Calcium intake, ↔ change in fat mass or percent body fat
Barr, 2007	Peripubertal girls (n=45)	↑ Calcium intake, ↔ body weight or body composition
Gunther *et al.,* 2007	Dortmund Nutritional and Longitudinally Designed Study (n=203)	↑ Dairy protein, ↑ BMI
Fiorito *et al.,* 2009	Girls (n=166)	↑ Milk intake, ↔ adiposity
Huh *et al.,* 2010	Project Viva (n=852)	↑ Milk or dairy, ↔ BMI and incident overweight

* For the associations listed, ↑ represents a positive association, ↓ represents a negative association and ↔ represents no association between the variables listed.

**Table 3:** Randomized Clinical Trials Examining the Effect of Milk or Milk Products on Body Weight and/or Body Composition in Children and Adolescents

Citation	Subjects	Treatments	Results*
Chan *et al.,* 1995	Girls (n=48)	Dairy (1200 mg calcium/d) vs. habitual intake	↑ Dairy, ↔ percent body fat and lean body mass
Cadogan *et al.,* 1997	Girls (n=80)	1 pint of whole or reduced fat milk vs. habitual intake	↑ Milk, ↔ body weight and body composition
Merrilees *et al.,* 2000	Girls (n=91)	Dairy supplementation (1000 mg calcium/d) vs. control	↑ Dairy, ↔ body weight and body composition
Volek *et al., * 2003	Boys (n=28)	Resistance training with either 3 serving milk/d or juice	↑ Milk, ↔ body composition and body fat
Lappe *et al.,* 2004	Girls (n=59)	Calcium rich diet (1500 mg/d) vs. habitual diet	↑ Calcium, ↔ body weight, BMI and body composition
Du *et al.,* 2004	Chinese girls (n=757)	Milk vs. milk + vitamin D vs. control	↑ Milk, ↑ body weight
Lau *et al.,* 2004	Chinese children (n=344)	Milk powder (40 g or 80 g) vs. control	↑ Milk powder, ↔ body weight and body fat
Albala *et al.,* 2008	Chilean children (n=98)	Milk supplementation (3 servings/d) vs. usual intake	↑ Milk, ↔ percent body fat ↑ Milk, ↑ lean body mass
Ghayour-Mobarhan *et al.,* 2009	Children (n=120)	-500 kcal diet with 2, 3, or 4 servings of dairy/d	Dairy food, ↔ BMI, body weight and body fat
St.Onge *et al.,* 2009	Children (n=45)	High (4 servings/d) vs. low (1 serving/d) milk intakes	↑ Milk, ↔ body weight and body composition
Kelishadi *et al.,* 2009	Children (n=95)	Isocaloric dairy diet (>800 mg calcium/d) vs. energy restricted diet vs. control	↑ Dairy, ↓ rise in BMI and waist circumference

* For the associations listed, ↑ represents a positive association, ↓ represents a negative association and ↔ represents no association between the variables listed.
